# A high‐temperature water vapor equilibration method to determine non‐exchangeable hydrogen isotope ratios of sugar, starch and cellulose

**DOI:** 10.1111/pce.14193

**Published:** 2021-09-30

**Authors:** Philipp Schuler, Marc‐André Cormier, Roland A. Werner, Nina Buchmann, Arthur Gessler, Valentina Vitali, Matthias Saurer, Marco M. Lehmann

**Affiliations:** ^1^ Research Unit of Forest Dynamics, Research Group of Ecosystem Ecology Swiss Federal Institute for Forest, Snow and Landscape Research WSL Birmensdorf Switzerland; ^2^ Department of Earth Sciences, Research Group of Ocean Biogeochemistry University of Oxford Oxford UK; ^3^ Department of Environmental Systems Science, Group of Grassland Sciences ETH Zurich Zürich Switzerland; ^4^ Department of Environmental Systems Science, Institute of Terrestrial Ecosystems ETH Zurich Zürich Switzerland

**Keywords:** growth, NSC, photoperiod, photosynthesis, secondary metabolism, δ^2^H

## Abstract

The analysis of the non‐exchangeable hydrogen isotope ratio (δ^2^H_ne_) in carbohydrates is mostly limited to the structural component cellulose, while simple high‐throughput methods for δ^2^H_ne_ values of non‐structural carbohydrates (NSC) such as sugar and starch do not yet exist. Here, we tested if the hot vapor equilibration method originally developed for cellulose is applicable for NSC, verified by comparison with the traditional nitration method. We set up a detailed analytical protocol and applied the method to plant extracts of leaves from species with different photosynthetic pathways (i.e., C_3_, C_4_ and CAM). δ^2^H_ne_ of commercial sugars and starch from different classes and sources, ranging from −157.8 to +6.4‰, were reproducibly analysed with precision between 0.2‰ and 7.7‰. Mean δ^2^H_ne_ values of sugar are lowest in C_3_ (−92.0‰), intermediate in C_4_ (−32.5‰) and highest in CAM plants (6.0‰), with NSC being ^2^H‐depleted compared to cellulose and sugar being generally more ^2^H‐enriched than starch. Our results suggest that our method can be used in future studies to disentangle ^2^H‐fractionation processes, for improving mechanistic δ^2^H_ne_ models for leaf and tree‐ring cellulose and for further development of δ^2^H_ne_ in plant carbohydrates as a potential proxy for climate, hydrology, plant metabolism and physiology.

## Abstract 2

Here, we present a hot water‐vapor equilibration method that is suitable for high‐throughput δ^2^H_ne_ analysis of sugar and starch. Additionally, we show first insights into differences of δ^2^H_ne_ values within and between C_3_, C_4_ and CAM plant species growing under the same constant climatic conditions.

## 1 INTRODUCTION

The isotopic composition of carbohydrates, which are the primary building blocks of plant biomass, is well known as a useful proxy for hydro‐climatic conditions and plant physiological processes that have occurred during their biosynthesis (Gaglioti et al., 2017; Gessler et al., 2014; Manrique‐Alba et al., 2020; McCarroll & Loader, 2004; Porter et al., 2014; Sass‐Klaassen et al., 2005; Saurer et al., 2012; Saurer, Borella, & Leuenberger, 1997). Various high‐throughput methods have been developed to study the carbon and oxygen isotopic composition of non‐structural plant carbohydrates (NSC; i.e., sugar and starch) (Lehmann et al., 2020; Richter et al., 2009; Wanek, Heintel, & Richter, 2001), and of structural carbohydrates such as tree‐ring or leaf cellulose (Boettger et al., 2007). In contrast, methods to investigate the non‐exchangeable hydrogen isotopic composition (δ^2^H_ne_) in plant carbohydrates are still mainly limited to cellulose (An et al., 2014; Arosio, Ziehmer, Nicolussi, Schlüchter, & Leuenberger, 2020; Epstein, Yapp, & Hall, 1976; Filot, Leuenberger, Pazdur, & Boettger, 2006; Mischel, Esper, Keppler, Greule, & Werner, 2015; Nakatsuka et al., 2020; Sauer, Schimmelmann, Sessions, & Topalov, 2009; Xia et al., 2020). Existing methods to analyse δ^2^H_ne_ values of NSC use site‐specific natural isotope fractionation nuclear magnetic resonance spectroscopy (NMR) or sample derivatization prior to isotope ratio mass spectrometry (IRMS) (Abrahim, Cannavan, & Kelly, 2020; Augusti, Betson, & Schleucher, 2008; Dunbar & Schmidt, 1984; Schleucher, Vanderveer, Markley, & Sharkey, 1999; Zhang, Quemerais, Martin, Martin, & Williams, 1994). These methods are, however, very laborious and limited by their sample throughput and/or produce explosive compounds that are difficult to work with. As a result, publications reporting δ^2^H_ne_ values of NSC are rare (Dunbar & Wilson, 1983; Ehlers et al., 2015; Luo & Sternberg, 1991). However, recent studies show the great potential of δ^2^H values of plant compounds to retrospectively determine hydrological and climatic conditions (Anhäuser, Hook, Halfar, Greule, & Keppler, 2018; Gamarra & Kahmen, 2015; Hepp et al., 2015, 2019; Sachse et al., 2012), as well as to disentangle metabolic and physiological processes (Cormier et al., 2018; Estep & Hoering, 1981; Sanchez‐Bragado, Serret, Marimon, Bort, & Araus, 2019; Tipple & Ehleringer, 2018) such as the proportional use of carbon sources (i.e., fresh assimilates vs. storage compounds) for plant growth (Lehmann, Vitali, Schuler, Leuenberger, & Saurer, 2021; Zhu et al., 2020). Enabling the analysis of δ^2^H_ne_ of NSC, especially sugar at the leaf level, will make it possible to study processes and environmental conditions which are shaping the ^2^H‐fractionation of carbohydrates at a much higher time resolution compared to the analysis of δ^2^H_ne_ of cellulose. New routines and high‐throughput analytical methods for δ^2^H_ne_ values of NSC are thus needed to enable widespread application in earth and environmental sciences.

The difficulty of establishing reliable methods for δ^2^H_ne_ values of NSC and cellulose is mainly caused by the presence of oxygen‐bound hydrogen atoms (H_ex_) that can freely exchange with hydrogen atoms of the surrounding liquid water and water vapor. The interference of H_ex_ greatly affects the analysis of δ^2^H_ne_, which retains useful information on climate, hydrology, metabolism and physiology. The oldest method of measuring δ^2^H_ne_ is to derivatize hydroxyl groups with nitrate esters, using a mixture of either H_2_SO_4_ or H_3_PO_4_ with HNO_3_ (Alexander & Mitchell, 1949; Boettger et al., 2007; DeNiro, 1981; Epstein et al., 1976). However, the nitration process requires a large sample amount, is labour intensive, uses hazardous derivatization reactions and leads to thermally unstable products. A newer derivatization method to measure δ^2^H_ne_ in sugars is using *N*‐methyl‐bis‐trifluoroacetamide to replace H_ex_ with trifluoroacetate derivatives, which are measured by gas chromatography ‐ chromium silver reduction/high‐temperature conversion‐IRMS (GC‐CrAg/HTC‐IRMS) (Abrahim et al., 2020). This method still relies on a large sample amount of >20 mg extracted NSC, a relatively long measuring time, and the limitation of measuring only one element per analysis. Potential alternative methods that work without derivatization and use smaller amounts of material are based on water vapor equilibration, which sets H_ex_ to a known isotopic composition that allows the determination of δ^2^H_ne_ by mass balance (Cormier et al., 2018; Filot et al., 2006; Sauer et al., 2009; Schimmelmann, 1991; Wassenaar & Hobson, 2000). However, established water vapor equilibration methods are mainly calibrated for analysis of δ^2^H_ne_ values of complex molecules such as cellulose, keratin and chitin (Schimmelmann et al., 1986; Wassenaar & Hobson, 2000) and whether these methods can also be used for the analysis of δ^2^H_ne_ in NSC remains to be shown. The main purpose of this study was therefore to establish a high‐throughput hot water vapor equilibration method to determine δ^2^H_ne_ of NSC, based on already established protocols for cellulose (Sauer et al., 2009). Nitration of cellulose and starch was additionally applied as an independent method to verify our results. Finally, we used the method to determine δ^2^H_ne_ values of NSC and cellulose extracted from leaves of plant species with different photosynthetic pathways (C_3_, C_4_ and CAM) grown under the same controlled climatic conditions.

## 2 MATERIALS AND METHODS

### 2.1 Cellulose, starch and sugar standards

As reference materials, we used both commercially available (*n* = 4; spruce cellulose, Fluka, Honeywell International Inc., Morristown, New Jersey, U.S.A., Prod. No. 22181; IAEA‐CH‐3, International Atomic Energy Agency (IAEA), Vienna, Austria; Merck cellulose (Cellulose native no. 2351, Merck, Darmstadt, Germany), Wei Ming (CYCLOCEL® Microcrystalline Cellulose, Wei Ming Pharmaceutical MFG. co., LTD., Taipei City, Taiwan), and in‐house produced cellulose standards (*n* = 5; Isonet, spruce, beech, Spain and Siberia), commercially available starch standards (*n* = 4; starch from maize, Fluka, Prod. No. 85652; starch from rice, Calbiochem, Merck KGaA, Darmstadt, Germany, Prod. No. 569380; starch from wheat, Fluka, Prod. No. 85649 and starch from potato, Merck, Prod. No. 1.01259.0250), commercially available standards for sugars of different classes (*n* = 6; sucrose, Merck, Prod. No. 1.07687; d‐(+)‐glucose ≥99.5%, SIGMA Life Science, St. Louis, Missouri, U.S.A., Prod. No. 49139; d‐(−)‐fructose ≥99%, Fluka, Prod. No. 47739; d‐(+)‐raffinose pentahydrate ≥99%, Fluka, Prod. No. 83400; d‐(+)‐trehalose dihydrate ≥99%, SIGMA Life Science, Prod. No. T9449 and myo‐Inositol ≥99.5%, Sigma Life Science, Prod. No. 57569) and two household sugars (Finish sucrose from 2019, Suomalainen Taloussokeri, Kantvik, Finland; Russian sucrose, household sugar from a Russian supermarket supplier). All reference materials were oven‐dried at 60°C for 48 hr and stored in an exicator at low relative humidity (2–5%) until further use.

### 2.2 Plant species, growing conditions and sampling

Ten plant species with different photosynthetic pathways grown under controlled conditions in walk‐in climate chambers (Bouygues E&S InTec Schweiz AG, Zurich, Switzerland) were used to apply the new method, and compare δ^2^H_ne_ of cellulose, starch and soluble sugars. The species selection covered C_3_ herbs and grasses (*Abelmoschus esculentus* (L.) Moench, *Cannabis sativa* L., *Hordeum vulgare* L., *Salvia hispanica* L. and *Solanum cheesmaniae* (L. Riley) Fosberg), C_4_ grasses (*Sorghum bicolor* (L.) Moench, *Zea mays* L.) and CAM plants (*Portulaca grandiflora* Hook., *Kalanchoe daigremontiana* Raym.‐Hamet & H.Perrier and *Phalaenopsis* Blume hybrid). Seeds or plantlets were sown or planted in 3 L pots containing potting soil (Kübelpflanzenerde, RICOTER Erdaufbereitung AG, Aarberg, Switzerland). The orchid *Phalaenopsis* was bought in a local supermarket and grown in a special substrate based on bark mulch. The climate chamber conditions were set to 16 daytime hours (30°C and 40% relative humidity), 8 nighttime hours (15°C and 60% relative humidity) and photosynthetically active radiation of 110 μmol m^−2^ s^−1^ at plant height with uniform fluorescent tubes (OSRAM L 36 W 777 Fluora, Osram Licht AG, Munich, Germany). All plants were regularly watered to field capacity with tap water (δ^2^H = −79.9 ± 2.4 ‰ during the experimental period) to avoid any water limitation, except for *Phalaenopsis* that was watered with 50 ml twice a week to keep the substrate moist but prevent root rot due to excess water. The plants were grown for three months to ensure ample leaf material was grown for harvest.

At the sampling day, three samples of fully developed mature leaves, each from individual plants or three pools of leaves of four plants in the case of *H. vulgare*, were sampled after 7 hr of light to allow the plants to synthesize enough sugars and starch on the day of harvest and to guarantee steady‐state leaf water enrichment conditions (Cernusak et al., 2016). The leaf samples were immediately transferred to gas‐tight 12 ml glass vials (‘Exetainer’, Labco, Lampeter, UK, Prod. No. 738W), stored on ice until the harvest was complete (≤2 hr), and then at −20°C in a freezer until further use (Appendix 1). The sample material was dried using a cryogenic water distillation method (West, Patrickson, and Ehleringer (2006), crumbled with a spatula (dicotyledon species) or cut with scissors (monocotyledon species) into small pieces, and 100 mg of the fragmented material was separated for cellulose extraction. The remaining leaf material was then ball‐milled to powder (Retsch MM400, Retsch, Haan, Germany) for NSC extraction.

### 2.3 Cellulose and starch nitration, and isotopic analysis of the nitrated products

Nitrates of cellulose and starch without exchangeable H were used as reference material to assess the δ^2^H_ne_ values derived from the hot water vapor equilibration method. Nitration of cellulose and starch standards was performed following the method of Alexander and Mitchell (1949), using a mixture of P_2_O_5_ and 90% HNO_3_. δ^2^H values of nitrated cellulose and starch were analysed with a TC/EA‐IRMS system, using a reactor filled with chromium as described by Gehre et al. (2015). Reference materials for δ^2^H measurements of cellulose and starch nitrates were the IAEA‐CH‐7 polyethylene foil (PEF; International Atomic Energy Agency, Vienna, Austria) for a first offset correction and the USGS62, USGS63 and USGS64 caffeine standards (United States Geological Survey, Reston, Virginia, U.S.A.) (Schimmelmann et al., 2016) for the final normalization.

All Isotope ratios (δ) are calculated as given in Equation (1) (Coplen, 2011):

(1) $$\delta =\frac{R_{\text{Sample}}-R_{\text{Standard}}}{R_{\text{Standard}}}$$

R = ^2^H/^1^H of the sample (R_Sample_) and of Vienna Standard Mean Ocean Water (VSMOW2; R_Standard_) as the standard defining the international isotope scale. To express the resulting δ values in permil (‰), results have been multiplied by 1,000.

### 2.4 Preparation of leaf cellulose and NSC for δ^2^H_ne_ analysis

Every compound (i.e., sugars, starch and cellulose) was extracted once per sample. Cellulose (hemicellulose) was extracted from 100 mg of the fragmented leaf material in F57 fibre filter bags (made up of polyester and polyethylene with an effective pore size of 25 μm; ANKOM Technology, Macedon NY, U.S.A.). In brief, the samples were washed twice in a 5% sodium hydroxide solution at 60°C, rinsed with deionized water, washed 3 times for 10 hr in a 7% sodium chlorite solution, which was adjusted with 96% acetic acid to a pH between 4 and 5, and subsequently rinsed with boiling hot deionized water, and dried overnight in a drying oven at 60°C. The neutral sugar fraction (‘sugar’, a mixture of sugars, typically glucose, fructose, sucrose and sugar alcohol [Rinne, Saurer, Streit, & Siegwolf, 2012]) were extracted from 100 mg leaf powder and further purified using ion‐exchange cartridges, following established protocols for carbon and oxygen isotope analyses (Lehmann et al., 2020; Rinne et al., 2012). This is needed to separate the sugar from other water‐soluble compounds such as amino acids which would alter the resulting δ^2^H_ne_ values (Schmidt, Werner, & Eisenreich, 2003). Starch was extracted from the remaining pellet of the sugar extraction via enzymatic digestion following the established method for carbon isotope analysis (Richter et al., 2009; Wanek et al., 2001). The same protocol was used to hydrolyse the commercial starch standards. Aliquots of the extracted sugar (including those derived from starch) were pipetted in 5.5 × 9 mm silver foil capsules (IVA Analysentechnik GmbH & Co. KG, Germany, Prod. No. SA76981106), frozen at −20°C, freeze‐dried, folded into cubes and packed into an additional silver foil capsule of the same type, folded again and stored in an exicator at low relative humidity (2–5%) until isotope analysis.

### 2.5 δ^2^H_ne_ analysis of cellulose and NSC using a hot water vapor equilibration method

One microgram of commercial starch or cellulose standard was packed into 3.3 × 5 mm silver foil capsules (IVA, Prod. No. SA76980506), which led to a total peak area between 20 and 30‐V seconds (Vs) of each IRMS analysis. For sugar standards, one mg was transferred first into a 5.5 × 9 mm silver foil capsule (IVA), and additionally packed in a second capsule of the same size and folded again. The reason for the double packing was the observation that sugar samples became liquefied and rinsed out of single‐packed capsules during the hot water vapor equilibration, which led to a loss of sample and to negative impacts on the analysis of δ^2^H_ne_ in sugars. Such rinsing was prevented by double packing and had no negative impact on the drying time of the sugars (Appendix 2). The double packing did not have a negative impact on the equilibration itself, as indicated by the high x_e_ of the sugars (Table 1). All packed samples were stored in an exicator at low relative humidity (2–5%) until isotope analysis.

**Table 1:** Results of the hot water vapor equilibrations of cellulose, sugars and starch (including the sugars derived from digested starch) of different classes and origins (referenced against PEF)

	Ref. material	δ^2^H_e1_ [‰]	SD_e1_	δ^2^H_e1_ [‰]	SD_e2_	x_e_ [%]	X_e.pot_ [%]	δ^2^H_ne_ [‰]	δ^2^H_nitro_ [‰]	δ^2^H_ne_‐δ^2^H_nitro_ [‰]	Rep.
**Cellulose**	**Isonet**	−57.1	1.1	−108.2	4.1	19.5	30.0	−42.2	−44.5	2.3	0.9
	**Beech**	−57.7	1.2	−114.3	3.3	19.5	30.0	−49.7	−50.8	1.2	1.0
	**Spruce**	−40.2	1.7	−96.3	3.3	19.4	30.0	−27.9	−30.7	2.7	1.1
	**Spain**	−49.8	0.8	−114.1	3.7	22.1	30.0	−33.4	−27.7	−5.7	N.A.
	**Siberia**	−164.5	2.2	−224.0	1.7	20.5	30.0	−184.3	−184.9	−0.6	N.A.
	**IAEA**	−65.1	1.0	−126.0	2.8	21.0	30.0	−58.2	−57.3	−0.9	1.4
	**Merck**	−63.5	1.0	−119.3	2.3	19.3	30.0	−56.9	−55.9	−1.0	1.0
	**Fluka**	−72.9	0.9	−120.3	3.0	16.4	30.0	−69.3	−50.5	−18.8	0.8
	**Wei Ming**	−67.0	1.8	−114.6	2.1	16.4	30.0	−62.3	−70.0	7.7	1.9
**Sugar**	**Finn. sucrose**	−133.5	3.7	−239.1	1.3	36.4	36.4	−157.8	N.A.	N.A.	5.8
	**Russ. sucrose**	−65.0	2.0	−169.7	2.2	36.1	36.4	−50.3	N.A.	N.A.	4.2
	**Merck sucrose**	−107.5	3.2	−214.2	1.7	36.8	36.4	−117.0	N.A.	N.A.	5.8
	**Glucose**	−31.3	2.2	−143.4	3.6	38.7	41.7	6.4	N.A.	N.A.	4.2
	**Fructose**	−47.6	2.9	−155.3	3.9	37.1	41.7	−21.9	N.A.	N.A.	4.9
	**Raffinose**	−16.4	1.6	−115.2	3.5	34.1	34.4	22.2	N.A.	N.A.	4.3
	**Trehalose**	−91.4	2.1	−196.1	3.3	36.1	36.4	−91.5	N.A.	N.A.	4.0
	**Myo‐Inositol**	−91.5	3.7	−246.6	7.7	53.5	50.0	−91.8	N.A.	N.A.	8.6
**Starch**	**Maize**	−32.9	1.2	−96.2	0.8	21.8	30.0	−16.6	−13.4	−3.1	N.A.
	**Maize starch hydrolysed**	−41.4	0.5	−132.7	1.8	31.5	41.7	−18.6	−13.4	−5.1	N.A.
	**Rice**	−71.6	2.0	−136.7	0.5	22.5	30.0	−65.9	−67.2	1.2	N.A.
	**Rice starch hydrolysed**	−76.2	1.1	−169.1	1.0	32.0	41.7	−69.2	−67.2	−2.0	N.A.
	**Wheat**	−58.4	2.1	−110.2	0.2	17.8	30.0	−51.3	−53.7	2.3	N.A.
	**Wheat starch hydrolysed**	−71.0	0.3	−162.9	0.2	31.7	41.7	−61.6	−53.7	−8.0	N.A.
	**Potato**	−127.1	1.8	−194.0	4.5	23.0	30.0	−137.9	−143.2	5.3	N.A.
	**Potato starch hydrolysed**	129.1	1.1	−221.8	3.7	32.0	41.7	−147.0	−143.2	−3.7	N.A.

*Note*: The following variables are given: δ^2^H_e1_ and δ^2^H_e2_ are average δ^2^H values in ‰ of all replicates and repetitions (cellulose and sugars as three independent repetitions with each water, each time in triplicates; starch and digested starch as one measurement per water with three replicates) with e_1_ representing the equilibration with water 1 and e_2_ representing the equilibration with water 2, SD_e1_ and SD_e2_ are the standard deviations of all repetitions (= precision), x_e_ depicts the proportion of exchanged hydrogen during the equilibration in % [Equation (2)], x_e.pot_ is the potential maximum proportion of exchangeable hydrogen based on the proportion of oxygen‐bound hydrogen in %, δ^2^H_ne_ represents the calculated δ^2^H of the non‐exchangeable hydrogen in ‰ [Equation (3)], δ^2^H_nitro_ denotes the δ^2^H of the corresponding nitrated compound in ‰, δ^2^H_ne_‐δ^2^H_nitro_ depicts the difference between δ^2^H_ne_ and δ^2^H_nitro_ in ‰ (= accuracy), Rep. = reproducibility, given as standard deviation between the resulting δ^2^H_ne_ of the three independent repetitions for cellulose and sugars in ‰, N.A., not available.

All samples were equilibrated in a home‐built offline equilibration system (Appendix 3), consisting of a heating oven with an in‐house designed equilibration chamber (Appendix 4) connected to a peristaltic pump (Gilson Incorporated, Middleton, USA). The equilibration chamber consisted of a sampler carousel (Zero Blank Autosampler, N.C. Technologies S.r.l., Milano, Italy) for solid samples with 50 cylindrical sample positions, where samples and reference materials could be placed, inserted into a cubic stainless steel chamber with a heat‐stable Viton ® O‐rings (Maagtechnic AG, Dübendorf, Switzerland, Prod. No. 15087359) surrounding the autosampler tray. The top of the chamber was sealed with a stainless steel metal plate using one stainless steel clamp at each corner. In the middle of the top metal plate, one inlet and one outlet connector were installed (Appendix 5). The inlet was connected to a stainless steel tube (i.e., feeding capillary, BGB, Switzerland), which was leading out of the oven where a santoprene pump tubing was fitted into a peristaltic pump (Appendix 6). The end of the santoprene pump tubing was inserted into a 50 ml falcon tube containing the equilibration water. The peristaltic pump provided a constant flow of the equilibration water (1.7 ml h^−1^) into the equilibration chamber. The temperature setpoint of the preheated oven was set to a constant 130°C, ensuring immediate evaporation of water after entering the equilibration chamber. The end of the outlet metal tube was inserted into a glass vessel and checked for vapor flow and condensation of the blown‐out vapor. After 2 hr of equilibration, the feeding capillary was switched to a capillary delivering dry nitrogen gas (N_2_ 5.0, PanGas AG, Dagmersellen, Switzerland, Prod No. 2220912) with a pressure of one bar for 2 hr to ensure complete removal of gaseous water in the chamber, which was still kept at 130°C. The duration of equilibration and drying, as well as the equilibration temperature, were step‐wise tested for cellulose to ensure maximum equilibration and no residual vapor. However, the high equilibration temperature of 130°C might be important to break down the crystalline structure of sugars and gelatinize starch to enable the access of water vapor (Gudasz, Soto, Sparrman, & Karlsson, 2020). For testing the reproducibility of the adapted method, triplicates of each type of cellulose and sugar samples were equilibrated independently on separate days following a standardized sample sequence (Appendix 7), in total three times with Water 1 (δ^2^H = −160‰) and three times with Water 2 (δ^2^H = −428‰). For starch and digested starch, triplicates were equilibrated only once with Water 1 and once with Water 2.

Subsequently, all samples (still hot) were immediately transferred into a Zero Blank Autosampler (N.C. Technologies S.r.l.), which was installed on a sample port of a high‐temperature elemental analyser system. The latter was coupled via a ConFlo III interface to a Delta^Plus^ XP IRMS (TC/EA‐IRMS, Finnigan MAT, Bremen, Germany). It is crucial to transfer the samples as fast as possible and still hot from the equilibration chamber to the autosampler to avoid any isotopic re‐equilibration of the sample with air moisture and water absorption. The autosampler carousel was evacuated to 0.01 mbar and afterwards filled with dry helium gas to 1.5 bar to avoid any contact with ambient water (vapor). The samples were pyrolysed in a reactor according to Gehre, Geilmann, Richter, Werner, and Brand (2004), and carried in a flow of dry helium (150 ml min^−1^) to the IRMS. Raw δ^2^H values of standard material (Table 1) were offset corrected using PEF standards (SD of PEF < 0.7‰ within one run).

Leaf sugar, starch and cellulose samples of three biological replicates were prepared as described above for the commercial standard material and equilibrated using identical settings. This corresponded to one equilibration with Water 1 and one with Water 2. Raw δ^2^H values of plant‐derived compounds were offset corrected using PEF. The calculated δ^2^H_ne_ of plant extracted sugar and sugar derived from starch (Table 2) were normalized against the δ^2^H_ne_ of Finnish, Russian and Merck sucrose from the method implementation (Table 1), while the calculated δ^2^H_ne_ of plant extracted cellulose were normalized against the δ^2^H values of the corresponding nitrocellulose of cellulose from spruce, Spain and Siberia.

**Table 2:** δ^2^H_ne_ values of plant‐derived sugar, starch and cellulose from leaf material

		δ^2^H_ne_ Starch [‰]		δ^2^H_ne_ Sugar [‰]		δ^2^H_ne_ Cellulose [‰]		Difference in δ^2^H_ne_ [‰]		
	Species	Mean	SD	Mean	SD	Mean	SD	Cell‐starch	Sugar‐starch	Cell‐sugar
**C**_**3**_	***Cannabis sativa***	−125.0	27.1	−99.4	15.9	−56.1	6.4	68.9	25.6	43.3
	***Solanum cheesmaniae***	−147.0	17.2	−99.4	6.9	−78.4	6.1	68.6	47.6	21
	***Salvia hispanica***	−133.9	23.3	−75.9	9.1	−50.0	18.1	83.8	58.0	25.8
	***Abelmoschus esculentus***	−126.1	12.3	−110.5	7.3	−63.4	10.6	62.7	15.5	47.1
	***Hordeum vulgare***	−76.7^a^	^a^	−74.8	5.1	−59.0	4.9	17.7	1.9	15.8
	**mean**	−121.7	20.0	−92.0	8.9	−61.4	9.2	60.3	29.7	30.6
**C**_**4**_	***Zea mays***	−60.6^a^	^a^	−44.8	2.6	−7.7	9.3	52.9	15.8	37.1
	***Sorghum bicolor***	−61.2^a^	^a^	−20.2	3.7	−25.3	6.7	35.9	41.0	−5.1
	**mean**	−60.9	^a^	−32.5	3.2	−16.5	8.0	44.4	28.4	16.0
**CAM**	***Portulaca grandiflora***	−24.8	33.7	−12.8	15.1	14.9	5.7	39.7	11.9	27.7
	***Kalanchoe daigremontiana***	−18.0	2.3	−13.2	3.6	−5.6	5.3	12.4	4.8	7.6
	***Phalaenopsis***	12.1^a^	^a^	44.2	2.2	23.3	1.2	11.2	32.1	−20.9
	**mean**	−10.2	18.0	6.0	6.9	10.9	4.0	21.1	16.3	4.8

*Note*: Plant species differing in photosynthetic pathways were grown under the same controlled conditions.

^a^ Due to low yields, starch samples of three replicates were pooled for *H. vulgare*, *Z. mays*, *S. bicolor* and *Phalaenopsis*, and thus could be only measured once.

### 2.6 Calculation of non‐exchangeable hydrogen isotope ratio (δ^2^H_ne_ )

According to Filot et al. (2006), the %‐proportion of exchanged hydrogen during the equilibrations [x_e_, Equation (2)] can be calculated as:

(2) $$x_{e}=\frac{\delta^{2}H_{e1}-\delta^{2}H_{e2}\;}{\alpha_{e-w}∙\left(\delta^{2}H_{w1}-\delta^{2}H_{w2}\right)}\;$$

where δ^2^H_e1_ and δ^2^H_e2_ are the δ^2^H values of the two equilibrated samples, δ^2^H_w1_ and δ^2^H_w2_ are the δ^2^H values of the two waters used, α_e‐w_ is the fractionation factor of 1.082 for cellulose (Filot et al., 2006). While α_e‐w_ needs to be adapted for different compounds and fractions with different functional groups (Schimmelmann, 1991), we consider α_e‐w_ of cellulose to be transferable to other carbohydrates as they all have the exchangeable hydrogen on hydroxyl groups. The fractionation factor we use in our method lies also within the range proposed in other studies (Schimmelmann, Lewan, & Wintsch, 1999; Wassenaar & Hobson, 2000).

δ^2^H_ne_ can then be calculated with Equation (3) using one of the two equilibrations (in this example equilibration with Water 1 [δ^2^H_e1_ and δ^2^H_w1_]):

(3) $$\delta^{2}H_{\mathrm{ne}}=\frac{\delta^{2}H_{e1}-x_{e}∙\alpha_{e-w}∙\delta^{2}H_{w1}-1000∙x_{e}∙\left(\alpha_{e-w}-1\right)\;}{1-x_{e}}$$

Statistical analyses (one‐way ANOVA and Tukey posthoc test) were performed using R version 3.6.3 (R.Core.Team, 2021).

## 3 RESULTS AND DISCUSSION

### 3.1 A hot water vapor equilibration method for determining δ^2^H_ne_ of sugar, starch and cellulose

Our in‐house implementation of the hot water vapor equilibration method for cellulose resulted in precise and accurate measurements of δ^2^H_ne_ values of cellulose (Table 1). δ^2^H_ne_ values of cellulose, ranging from −44.5 to −70.0‰, were measured with high precision as indicated by the standard deviations (SD_e1_ and SD_e2_) ranging between 0.9‰ and 4.1‰ for both equilibration waters. In addition, high accuracy was found, as indicated by a deviation of −1.0 to +5.7‰ between the δ^2^H_ne_ value of the hot water vapor equilibration and the δ^2^H value of the corresponding cellulose nitrate (δ^2^H_ne_‐ δ^2^H_nitro_), except for two of the commercial cellulose samples from Fluka and Wei Ming, with a deviation of −18.8 and +7.7‰, respectively. For the samples with high accuracy, the calculated x_e_ ranged between 19.3 and 22.1% compared to a theoretical x_e.pot_ of 30%. These x_e_ values are comparable to those 20.5 ± 0.1% observed in the original implementation of the hot water vapor equilibration for cellulose (Sauer et al., 2009). For the two samples with low accuracy, x_e_ reached only 16.4%. The reason for the low x_e_ and the resulting low accuracy of the commercial cellulose from Fluka and the Wei Ming remains elusive. Tentatively, it could be explained by a different extraction method and purification of these cellulose samples, leading to different nanostructures (Jungnikl, Paris, Fratzl, & Burgert, 2007) or particle sizes, which in turn leads to different accessibility of water vapor to the cellulose molecule (Chami Khazraji & Robert, 2013). Nevertheless, the results show that the hot water vapor equilibration is suitable to determine δ^2^H_ne_ with high accuracy and precision if the principle of identical treatment (Werner & Brand, 2001) is applied, that is, all samples are prepared and measured in the same way. Besides, the calculated x_e_ values of the IAEA‐CH‐7 reference material without any H_ex_ were close to 0 throughout all measurements, denoting the absence of absorbed water on the surface of each compound, as well as the analytical reproducibility for all δ^2^H_ne_ values of cellulose, was high as indicated by a standard deviation of 0.8 to 1.9‰ for three repetitions.

The same method was also applied to analyse δ^2^H_ne_ of NSC (Table 1). δ^2^H_ne_ values of sugars of different classes, ranging from 6.4 to −157.8‰, were also measured with high precision as indicated by an SD ranging between 1.3 and 7.7‰ for both equilibration waters, which is comparable to the precision of derivatization methods (Dunbar & Schmidt, 1984: 1.9‰; Augusti et al., 2008: 2 and 10‰; Abrahim et al., 2020: 0.4 and 3.6‰). As no nitrated sugars were available due to the safety problems with sugar nitration, we could not calculate the accuracy. We, however, can assume that the accuracies for sugars should be in a comparable range as those derived from digested starch (−8.0 and −2.0‰). The reproducibility of the results for all tested commercial sugars ranged between 4.0 and 8.6‰ for three repetitions. The x_e_ of the different sugars ranged between 34.1 and 53.5% and was thus similar or very close to x_e.pot_, which gives further confidence in the reliability of the method for sugars. The smaller deviation of x_e_ from x_e.pot_ for sugars than for cellulose might be explained by the dissolution of the sugars during the hot water vapor equilibration, leading to a breakdown of the crystal structure of the sugars. This might have facilitated a complete exchange of H_ex_ with the water vapor in sugars, that is, not feasible for cellulose (Sauer et al., 2009; Schimmelmann, 1991).

The δ^2^H_ne_ of equilibrated but undigested starch was close to the δ^2^H_ne_ of the nitrated starch, measured with a precision ranging between 0.2 to 4.5‰ and accuracy between −3.1 and +5.3‰. The x_e_ of the undigested starch was between 17.8 and 23.0%, and thus comparable to the results derived from cellulose. For digested starch, the precision ranged from 0.3 to 3.7‰ and the accuracy between −2.0 and −8.0‰. The x_e_ of the digested starch ranged between 31.5 and 32.0% and was thus lower than the measured x_e_ (38.7%) and x_e.pot_ of pure glucose (41.7%). This lower x_e_ of starch‐derived sugar compared to glucose could be explained by incomplete digestion of the starch to glucose monomers, leading to a mixture of mono‐ and oligosaccharides.

Overall, our results show that sugars of different classes, as well as sugar derived from digested starch, can be measured with high precision, accuracy and reproducibility. On a daily routine, we were able to measure up to 66 NSC samples and 32 standards. This proves that a method is now a reliable tool that enables high‐throughput analysis of δ^2^H_ne_ of NSC in plants or in other environmental or biological samples.

### 3.2 Application of the method for analysis of δ^2^H_ne_ in plant‐derived compounds

**Figure 1:**
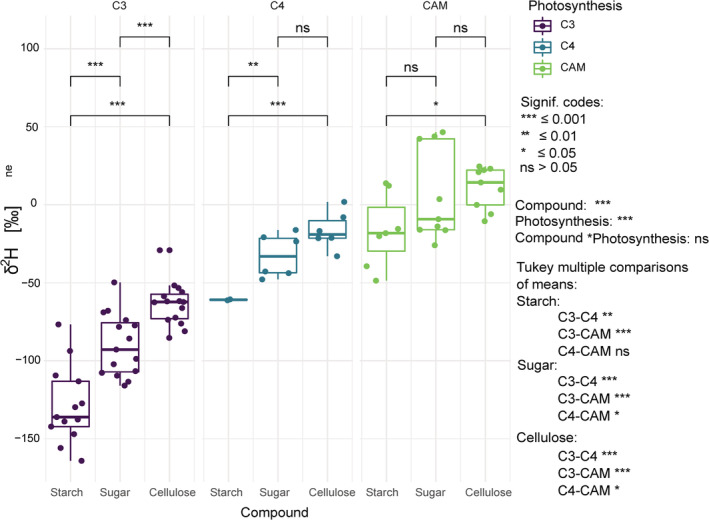
Comparison of δ^2^H_ne_ between starch, sugar and cellulose of leaves within and between the three photosynthesis types. The boxplots show the estimated significance levels using a linear model comparing the compounds within the photosynthesis types. On the low‐right side, the significant levels of a Tukey posthoc test comparing the photosynthesis types for all three compounds are given [Colour figure can be viewed at wileyonlinelibrary.com]

The analyses of non‐exchangeable hydrogen in sugar, starch and cellulose extracted from leaves of the plants grown in a climate chamber under controlled conditions showed strong differences (Figure 1, Table 2). Generally, among all the plant species and photosynthesis pathway types, starch was the most ^2^H‐depleted compound, followed by sugar, while cellulose was the most ^2^H‐enriched compound. In C_3_ plants, all compounds were significantly different from each other and showed the strongest ^2^H‐depletion of all photosynthetic types, with a mean δ^2^H_ne_ of −121.7‰ for starch, −92.0‰ for sugar and −61.4‰ for cellulose. In C_4_ plants, mean δ^2^H_ne_ values of −60.9‰ for starch were significantly lower compared to those of −32.5‰ and −16.5‰ for sugar and cellulose and thus reflect intermediate δ^2^H_ne_ values compared to C_3_ and CAM plants. In CAM plants, only δ^2^H_ne_ values of starch and cellulose differed significantly and showed the strongest ^2^H‐enrichment of all photosynthetic types, with a mean δ^2^H_ne_ of −10.2‰ for starch, 6.0‰ for sugar and 10.9‰ for cellulose. The comparison of the δ^2^H_ne_ of the same compound between the photosynthetic types resulted in significant differences between C_3_ and C_4_ and between C_3_ and CAM plants. The difference in sugar and cellulose between C_4_ and CAM plants was only slightly significant and not significant for starch. Our results go along with studies on δ^2^H_ne_ values of organic matter and cellulose, showing also a ^2^H‐enrichment in C_4_ and CAM plants compared to C_3_ plants (Leaney, Osmond, Allison, & Ziegler, 1985; Sternberg, Deniro, & Ajie, 1984). While the observed variation in δ^2^H_ne_ of NSC and cellulose among the photosynthetic pathways are unlikely to be explained solely by differences in leaf water ^2^H enrichment (Kahmen, Schefuß, & Sachse, 2013; Leaney et al., 1985), higher leaf water δ^2^H values might partially contribute to higher δ^2^H_ne_ of NSC and cellulose in CAM plants compared to C_3_ plants (Smith & Ziegler, 1990). Thus, δ^2^H measurement of leaf water would be important to disentangle the photosynthetic ^2^H‐fractionation from leaf water to leaf NSC and cellulose within and between the photosynthetic types. However, δ^2^H_ne_ difference among photosynthetic pathways and compounds are likely explained by ^2^H‐fractionations in biochemical pathways, including the usage of cytoplasm derived malate as a proton source and glucose precursor in CAM and C_4_ plants (Yamori, Hikosaka, & Way, 2014; Zhou et al., 2018), which might overlay the signal of the strongly ^2^H‐depleted NADPH produced via photosystem II (Luo, Steinberg, Suda, Kumazawa, & Mitsui, 1991). In summary, the analyses of δ^2^H_ne_ in sugars, starch and cellulose might be used to generally distinguish plants with C_3_, C_4_ and CAM photosynthesis.

Above that, δ^2^H_ne_ values in CAM plants may indicate if a facultative CAM plant performs C_3_ or C_4_ photosynthesis in the absence of water stress (Guralnick, Gilbert, Denio, & Antico, 2020; Winter, Garcia, & Holtum, 2008). The higher the contribution of C_3_ or C_4_ photosynthesis to a CAM plant's total carbon dioxide fixation, the more depleted are the δ^2^H_ne_ values of cellulose and NSC (Luo & Sternberg, 1991; Sternberg, Deniro, & Johnson, 1984), thus indicating the absence of water stress. Among all the tested plant species, the orchid *Phalaenopsis* was the only species with a positive δ^2^H_ne_ value in all compounds, and thus likely the only species with no or only a negligible amount of C_3_ photosynthesis in mature leaves. However, the observation that *Phalaenopsis* sugars are more ^2^H‐enriched than cellulose in mature leaves could be explained by the presence of C_3_ photosynthesis in the developing leaves (Guo & Lee, 2006), leading to ^2^H‐depleted cellulose during leaf formation. For the other two CAM species, the C_3_ or C_4_ photosynthesis contributed a higher fraction to the total carbon dioxide fixation due to the absence of water limitation and thus had lower δ^2^H_ne_ values for NSC and cellulose.

The generally lower δ^2^H_ne_ values of NSC compared to cellulose (Table 2) can be explained by the ^2^H‐depletion during photosystem II NADPH formation and the subsequent transfer of the ^2^H‐depleted H during the reduction of glyceraldehyde‐3‐phosphate, continuous enzymatic H‐exchange between carbohydrates and water and kinetic isotope effects during metabolic processes (Cormier et al., 2018; Cormier, Werner, Leuenberger, & Kahmen, 2019). Our results are supported by a previous study (Luo & Sternberg, 1991; Schleucher et al., 1999), showing that nitrated starch was more ^2^H‐depleted than nitrated cellulose within the same autotrophic photosynthetic tissue, which can be interpreted as another proof for the reliability of the new method for δ^2^H_ne_ values of NSC. The high variability in ^2^H‐fractionation in the sequence from sugars to starch to cellulose (Table 2) between all tested species indicates high variability in common ^2^H‐fractionation processes, which is also supported by recent studies (Cormier et al., 2018; Sanchez‐Bragado et al., 2019). Thus, the variability in ^2^H‐fractionation may find application in future plant physiological studies, investigating stress responses or short‐ and long‐term carbon dynamics. We assume that δ^2^H_ne_ of NSC are susceptible to diel or seasonal changes in environmental conditions such as temperature and light intensity due to their short turnover time (Fernandez et al., 2017; Gibon et al., 2004). The variability in ^2^H‐fractionation between different species might also be important if multiple tree species are used during the establishment of tree‐ring isotope chronologies in dendroclimatological studies (Arosio, Ziehmer‐Wenz, Nicolussi, Schlüchter, & Leuenberger, 2020).

In conclusion, we show that a hot water vapor equilibration method originally developed for cellulose can be adapted for accurate, precise and reproducible analyses of δ^2^H_ne_ in non‐structural carbohydrates (NSC) such as sugar and starch. By applying the method for compounds from different plant species, we demonstrated that this analytical method can now be used to estimate ^2^H‐fractionation among structural and NSC and to distinguish plant material from plants with different photosynthetic pathways. It should be noted that the method presented herein enables analysis of δ^2^H_ne_ of bulk sugar and sugar derived from digested starch and is therefore not compound‐specific nor position‐specific. Yet, our δ^2^H_ne_ method allows us to measure NSC samples in high‐throughput and we thus expect that it will help to identify important ^2^H‐fractionation processes. These findings could then eventually be studied in more detail with compound‐specific methods (GC‐IRMS [Abrahim et al., 2020]) or methods giving positional information (NMR [Ehlers et al., 2015]). We therefore expect that the method will find widespread applications in plant physiological, hydrological, ecological and agricultural research to study NSC fluxes and plant performance, and the beverage and food industry, to distinguish between sugars of different origins, which could be applied to check if a certain product is altered by the addition of low‐cost supplements. We also expect that the method can help to improve mechanistic models for ^2^H distributions in organic material (Roden, Lin, & Ehleringer, 2000; Yakir & DeNiro, 1990). The method may further help, in combination with other hydrogen isotope proxies (e.g., fatty acids, n‐alkanes or lignin methoxy groups), researchers to better understand metabolic pathways and fluxes, shaping the hydrogen isotopic composition of plant material.

## CONFLICT OF INTEREST

The authors declare no conflicts of interest.

## Supporting information


**Appendix S1.** Supporting Information.

## Data Availability

The data that support the findings of this study are available from the corresponding author upon reasonable request.
