# Supplementary material for: A high‐temperature water vapor equilibration method to determine non‐exchangeable hydrogen isotope ratios of sugar, starch and cellulose
Source: Plant Cell Environ. 2021 Sep 30;45(1):12–22. doi: 10.1111/pce.14193 (PMC9291759; doi:10.1111/pce.14193)
Supplement: Supplementary file 1 — Appendix S1. Supporting Information. [file PCE-45-12-s001.docx]

**Supplementary**


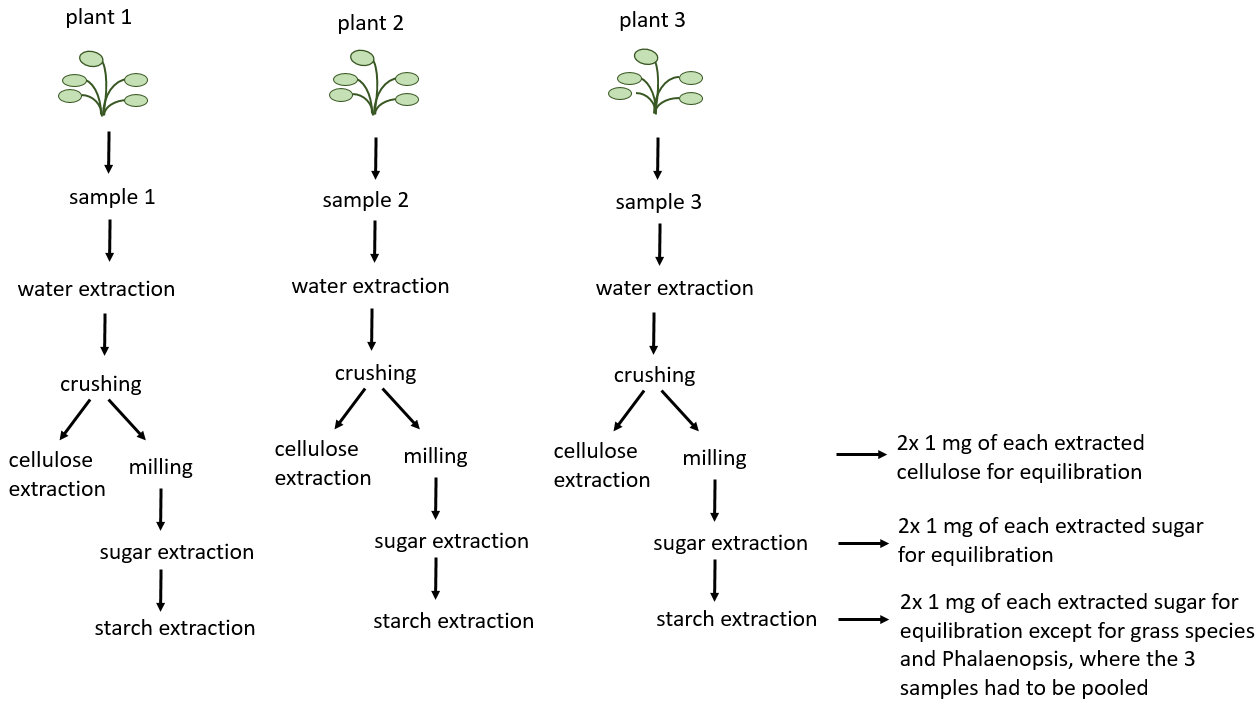


Appendix 1: Overview of the sample processing of the plant derived samples. For *H. vulgare*, the plant 1, 2 and 3 representing individual pools of four plants.

Appendix 2: δ^2^H measurement of three sugars measured after two, four, and six hours drying with dry nitrogen gas after equilibration


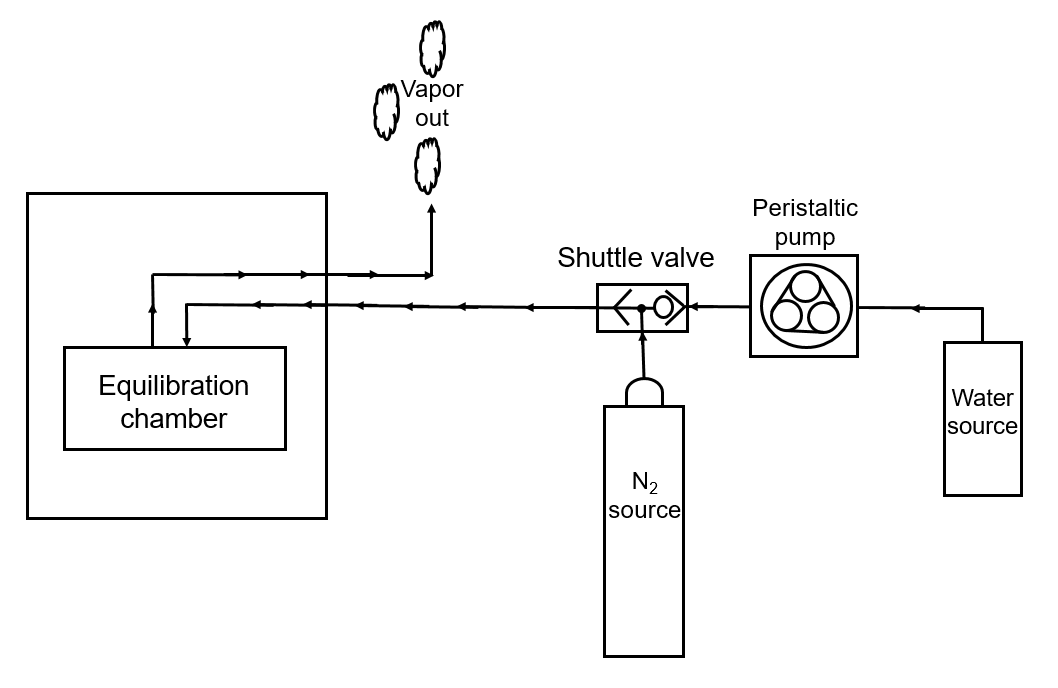


Appendix 3: Overview sketch of the equilibration system


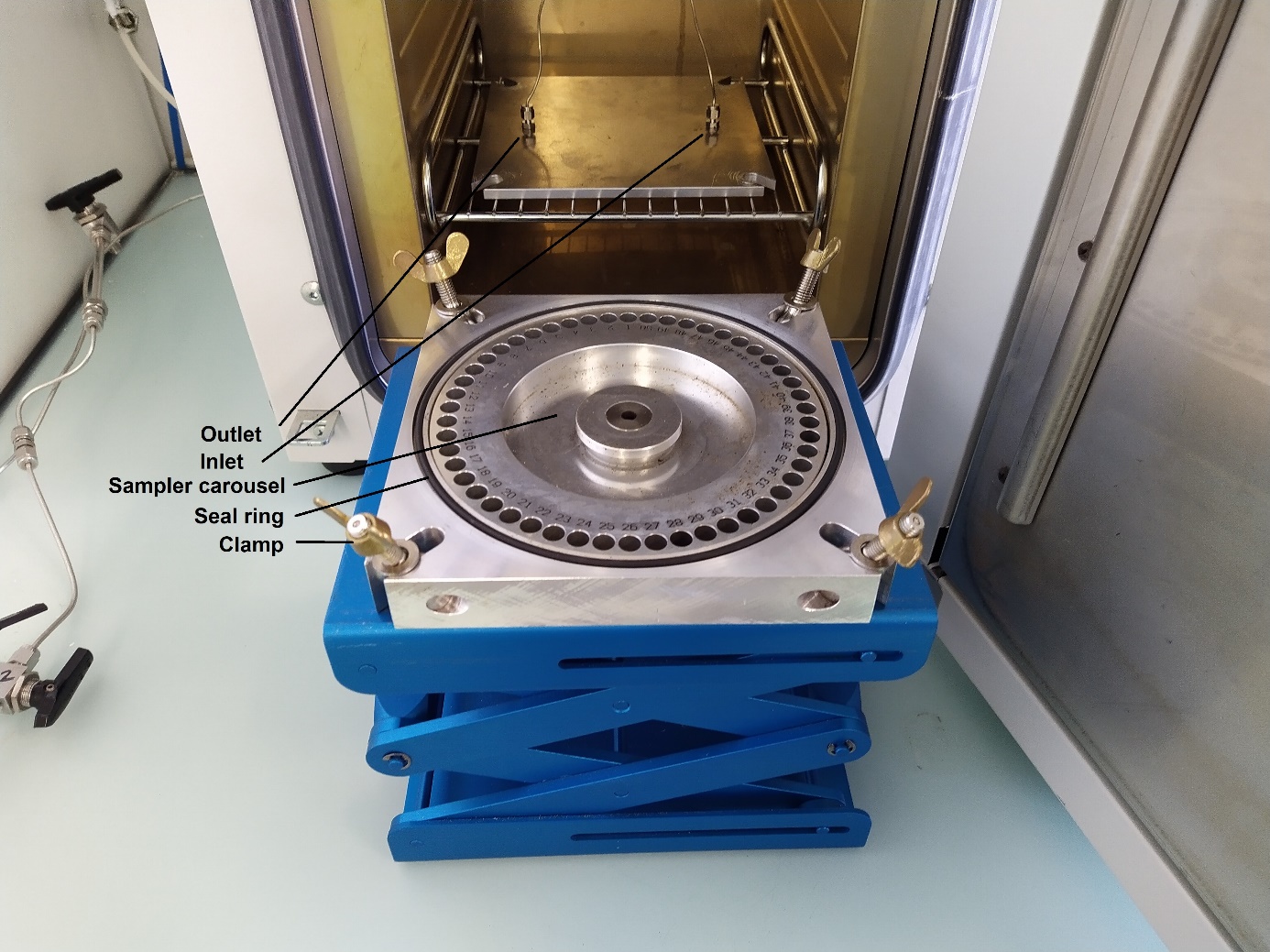


Appendix 4: Inner structure of the equilibration chamber


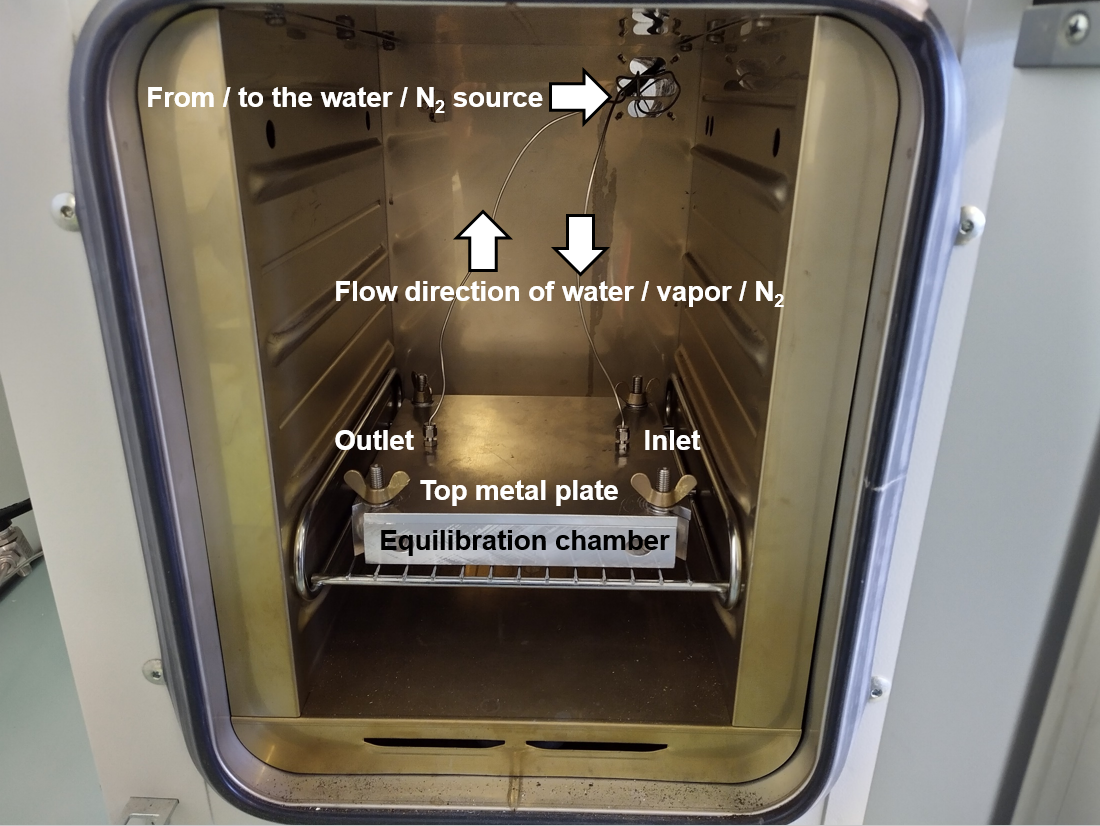


Appendix 5: Outer structure of the equilibration chamber


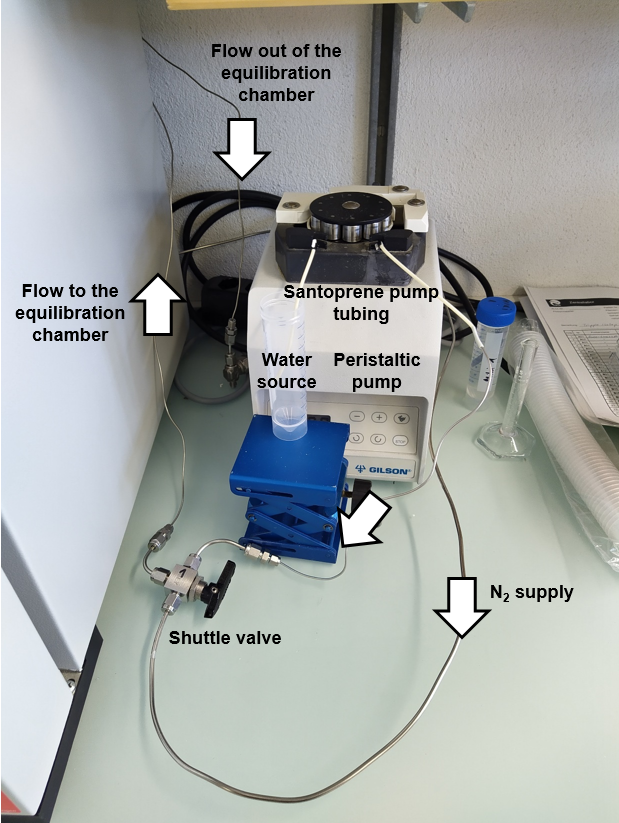


Appendix 6: Water source and dry nitrogen gas connection


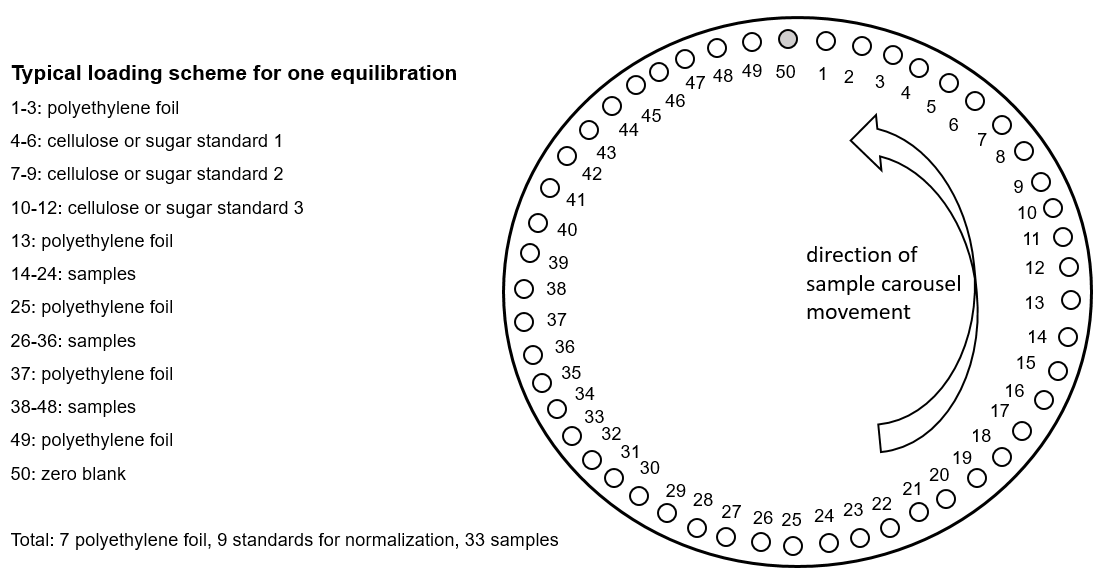


Appendix 7: Typical loading scheme for one hot water vapor equilibration
